# User-Centered LLM Chatbot to Bridge Information Gaps in Retirement Community

**DOI:** 10.1093/geroni/igaf122.4230

**Published:** 2025-12-31

**Authors:** Amy Deng, Kaylee Zhang, Ethan Liu, Ryker Hou, George Choi, Xingyu Li, Feng Chen

**Affiliations:** University of Washington, Seattle, Washington, United States; University of Washington, Seattle, Washington, United States; University of Washington, Seattle, Washington, United States; University of Washington, Seattle, Washington, United States; University of Washington, Seattle, Washington, United States; University of Washington, Seattle, Washington, United States; University of Washington, Seattle, Washington, United States

## Abstract

Lack of technology and eHealth literacy limits many retirement-community residents from retrieving essential daily information and engaging with online resources. Existing intranet portals often use small text, complex menus, and keyboard input that clash with age-related changes in vision, dexterity, and search self-confidence. To tackle these barriers, we partnered with older adults to co-design a large-language-model (LLM) powered conversational assistant specially designed for retirement community residents. Iterative interviews, observation sessions, and sketch workshops produced three personas capturing diverse goals and backgrounds. These personas guided the inclusion of large print and high contrast themes, adjustable font and color palettes, voice-to-text input, and one-tap example prompts. In parallel, we tested multiple LLMs (GPT-3.5-Turbo, GPT-4.1, Gemini 2.0, Llama 3.3-70B) with elder-centered prompts and selected GPT-4.1 for deployment based on superior completeness and clarity. Qualitative usability sessions revealed that participants valued immediate formatting controls and hands-free interaction. They conclude that the developed chatbot is friendly and easy to navigate, but also point out the need for transparent source links for reassurance. Participants reported greater confidence when seeking technology tips or health information after short use, suggesting early gains in digital self-efficacy. Collectively, the findings show that an LLM assistant grounded in persona-driven, user-centered design can enhance perceived accessibility, confidence, and efficiency for older adults while illuminating refinements, such as audio read-back and family-mediated configuration, needed for sustained adoption. Future work will examine longitudinal engagement and community-wide rollout to verify the impact of the chatbot.

